# Development of A Chimeric Antigen Receptor Targeting C-Type Lectin-Like Molecule-1 for Human Acute Myeloid Leukemia

**DOI:** 10.3390/ijms18112259

**Published:** 2017-10-27

**Authors:** Eduardo Laborda, Magdalena Mazagova, Sida Shao, Xinxin Wang, Herlinda Quirino, Ashley K. Woods, Eric N. Hampton, David T. Rodgers, Chan Hyuk Kim, Peter G. Schultz, Travis S. Young

**Affiliations:** 1 Department of Biology, California Institute for Biomedical Research (Calibr), La Jolla, CA 11119, USA; elaborda@calibr.org (E.L.); magdalena.mazagova@vrtx.com (M.M.); xwang@poseida.com (X.W.); hquirino@calibr.org (H.Q.); awoods@calibr.org (A.K.W.); ehampton@calibr.org (E.N.H.); drodgers@regulusrx.com (D.T.R.); 2 Department of Chemistry and The Skaggs Institute for Chemical Biology, The Scripps Research Institute, La Jolla, CA 11119, USA; sidashao@scripps.edu; 3 Department of Biological Sciences, Korea Advanced Institute of Science and Technology (KAIST), Daejeon 34141, Korea

**Keywords:** AML, CAR-T-cell, CLL-1, hematopoiesis, optimization

## Abstract

The treatment of patients with acute myeloid leukemia (AML) with targeted immunotherapy is challenged by the heterogeneity of the disease and a lack of tumor-exclusive antigens. Conventional immunotherapy targets for AML such as CD33 and CD123 have been proposed as targets for chimeric antigen receptor (CAR)-engineered T-cells (CAR-T-cells), a therapy that has been highly successful in the treatment of B-cell leukemia and lymphoma. However, CD33 and CD123 are present on hematopoietic stem cells, and targeting with CAR-T-cells has the potential to elicit long-term myelosuppression. C-type lectin-like molecule-1 (CLL1 or CLEC12A) is a myeloid lineage antigen that is expressed by malignant cells in more than 90% of AML patients. CLL1 is not expressed by healthy Hematopoietic Stem Cells (HSCs), and is therefore a promising target for CAR-T-cell therapy. Here, we describe the development and optimization of an anti-CLL1 CAR-T-cell with potent activity on both AML cell lines and primary patient-derived AML blasts in vitro while sparing healthy HSCs. Furthermore, in a disseminated mouse xenograft model using the CLL1-positive HL60 cell line, these CAR-T-cells completely eradicated tumor, thus supporting CLL1 as a promising target for CAR-T-cells to treat AML while limiting myelosuppressive toxicity.

## 1. Introduction

AML is the most common type of acute leukemia in adults. Established first line and consolidation chemotherapy is effective for 70–80% of patients; however, with a five-year survival rate of 26%, the development of new therapies is urgently required [1]. The genetic engineering of patient-derived T-cells to express chimeric antigen receptor (CAR) that enables the major histocompatibility complex (MHC)-independent targeting of malignant cells has been widely successful in the treatment of B-cell leukemia. CAR-T-cells specific for CD19 have demonstrated complete and durable remissions in 70–90% of Acute Lymphoblastic Leukemia (ALL) patients that have failed multiple lines of prior therapy [2]. Due to the immunosuppressive nature of AML, the activation of the immune system through genetically engineered T-cell therapy presents a promising, curative option for patients [2,3].

To translate the promising results achieved for CAR-T-cell therapy in ALL to AML, several groups have developed CAR-T-cells targeting, among others, CD33 and CD123 [4,5,6,7,8]. However, because CD33 and CD123 are also expressed on hematopoietic stem cells (HSCs), these CAR-T-cells have the potential to cause long-term myeloablation, paradoxically due to CAR-T-cells’ potency and persistence. Indeed, a recent fatal event in a clinical trial of allogeneic CAR-T-cells targeting CD123 [9], severe pancytopenia in an AML patient treated with anti-CD33 CAR-T-cells [10], and impaired hematopoiesis in xenograft models of human hematopoiesis have been described [4,5]. In addition, the high heterogeneity of AML calls for the development of CAR-T-cells that will enable the treatment of a broader patient population with reduced risk of long-term adverse effects.

Here, we designed and optimized a CAR-T-cell targeting the type II transmembrane glycoprotein, CLL1, a target recently used for cancer immunotherapies [11,12] that is expressed in 92% of AML patients [13]. CLL1 is highly restricted to the myeloid lineage and is absent in the CD34+CD38− HSC compartment [13]. Moreover, CLL1 is present on a small population of chemotherapy-resistant leukemic stem cells hypothesized to be responsible for relapse [14,15], making it an ideal target antigen for the treatment of AML patients with CAR-T-cells [12,13,15].

## 2. Results and Discussion

**Figure 1:**
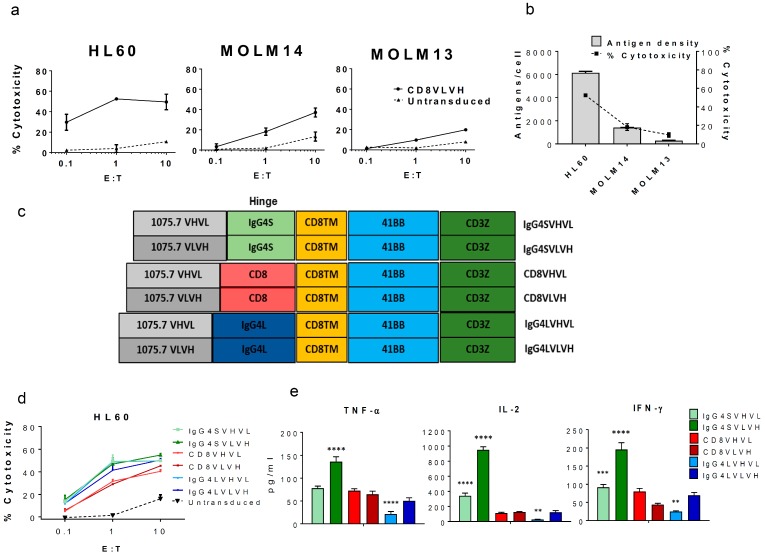
Validation of C-type lectin-like molecule-1 (CLL1) as a target for acute myeloid leukemia (AML) and the optimization of CLL1 chimeric antigen receptor (CAR) engineered T-cells (CAR-T-cells). CLL1 antigen is a valid AML target for CAR-T-cell therapy. (**a**) Anti-CLL1 CAR-T construct (CD8VLVH) cytotoxicity on AML cell lines at different Effector to Target (E:T) ratios. Data represents the mean values of triplicates +/− standard deviation (SD). (**b**) The percentage of cytotoxicity (E:T = 1) induced by anti-CLL1 CAR-T (CD8VLVH) strongly correlates with target cell antigen density. Antigen density was determined using a BD Quantibrite™ Beads PE Fluorescence Quantitation Kit. (**c**) Schematic of the CAR constructs designed and lentivirally transduced into healthy-donor derived activated T-cells (average transduction 50%). All constructs were sorted to normalize CAR expression prior to assays (final CAR expression: 90–98%). (**d**) In vitro cytotoxicity of each construct against the AML cell line HL60 at indicated E:T ratios. (**e**) Cytokine production for each construct from the cytotoxicity assay shown in (**d**) with E:T = 1:1. Data represents the mean values of triplicates +/− SD. Results shown are representative of two different donors. ** *p* ≤ 0.01, *** *p* ≤ 0.001, **** *p* ≤ 0.0001 compared to CD8VLVH, one-way ANOVA, and Dunnet’s multiple comparison post-test.

To develop a CAR that targets CLL1, we used the scFv from the anti-CLL1 monoclonal antibody 1075.7 (Kd = 0.36 nM, IgG) [15]. We have previously used this clone in a bispecific antibody format with promising results [12]. Compared to bispecific antibody-based immunotherapies, more potent antitumor responses are expected due to the costimulatory domains genetically engineered into the T-cells. Initially, we combined 1075.7 scFv (light chain (VL) preceding the heavy chain (VH)) with the CD8 hinge-transmembrane, 4-1BB co-stimulatory, and CD3zeta domains used in the well-studied anti-CD19 CAR-T (CTL019) [16]. Importantly, Tashiro and colleagues recently compared the cytolytic activity and cytokine release of different costimulatory domains on a CAR targeting CLL1, and pointed to 41BB as the most potent construct [11]. Herein, the CD8VLVH construct was transduced into healthy donor-derived human T-cells using lentivirus, as previously described [17]. These cells demonstrated antigen-specific killing of CLL1-positive AML cell lines HL60, MOLM14, and MOLM13 in vitro (Figure 1a), with potency that correlated with antigen density (Figure 1b).

The design of the hinge region (also known as the spacer domain) which separates the scFv from the transmembrane domain has been previously shown to be important in CAR-T-cell activity by controlling the distance of the immunological synapse between the CAR-T-cell and the target cell [17,18,19]. To empirically determine the optimal length for the anti-CLL1 CAR, we replaced the 45-amino acid CD8 hinge with longer or shorter variants derived from the human IgG4 hinge. The long hinge consisting of 230 amino acids from IgG4 CH2 and CH3 domains (IgG4L) and the short hinge (IgG4S) was derived from the 12 amino acids of the IgG4 hinge region with an S228P mutation, which we and others have shown to promote inter-chain disulfide bond formation [17,20]. Each hinge construct was tested with the 1075.7 scFv in VHVL or VLVH formats (Figure 1c). Candidate constructs were transduced individually into T-cells and sorted to normalize CAR expression (Figure S1). Both IgG4 hinges and scFv orientations induced higher cytotoxicity in HL60 compared to CD8 hinge-based CARs (Figure 1d); interestingly, the IgG4S hinge with VLVH scFv orientation secreted the greatest amounts of interferon (IFN)-γ, tumor necrosis factor (TNF)-α, and interleukin (IL)-2, over other forms (Figure 1e). This is likely due to the decreased distance between the target and the CAR-T-cell created by the shorter hinge, a trend previously reported for CAR constructs [20]. Other biophysical properties of the hinge, i.e. the rigidity, could be relevant for the CAR design, affecting the killing ability of CAR-T-cells, and will need further investigation. Importantly, cytokine production has been associated with superior in vivo activity for CAR-T-cells [21]. Based on these results, the IgG4SVLVH anti-CLL1 CAR-T construct was used in all further studies.

**Figure 2:**
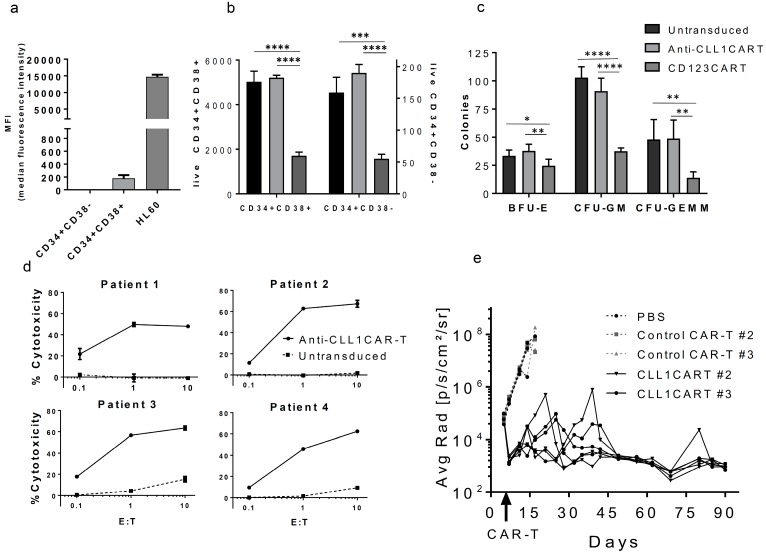
Anti-CLL1 CAR-T efficiently targets AML blasts in vitro and eradicates HL60 tumors in vivo while sparing hematopoiesis. Anti-CLL1 CAR-T (IgG4SVLVH) does not affect hematopoietic stem cell colony formation while inducing robust cytotoxicity on patient-derived AML blasts in vitro, and eradicates HL60 xenografts in vivo. (**a**) CLL1 Median Fluorescence Intensity (MFI) of cord-blood derived CD34+CD38− and CD34+CD38+ subsets. Results are representative of two different donors. Background MFI from an unstained control was subtracted from all test samples. (**b**) Anti-CLL1 CAR-T cytotoxicity on CD34+CD38− and CD34+CD38+ cells. Freshly isolated CD34+ cells were incubated with CAR-T-cells labeled with PKH67 at E:T = 10:1. After 4 h, cells were stained with anti-CD34, anti-CD38, and 7-AAD, and count bright absolute counting beads (Thermo Fisher Sci., Waltham, MA, USA) were added prior to acquisition by flow cytometry. (**c**) Colony-Forming Unit assay (CFU) of Hematopoietic Stem Cell (HSC) co-incubated for 4 h with CAR-T-cells. HSC were isolated and cultured with CAR-T-cells as per (**b**). The mixture was plated (500 HSC/plate) in semi-solid media and CFU were scored for each condition. For (**a**–**c**) data represent the mean values of triplicates +/− SD of two different donors. *p ≤ 0.05, **p ≤ 0.01, ***p ≤ 0.001, ****p ≤ 0.0001, one-way ANOVA, Tukey’s multiple comparison post-test. BFU-E: burst-forming unit-erythroid; CFU-GM: granulocyte/macrophage progenitor cells; CFU-GEMM: granulocyte, erythroid, macrophage, megakaryocyte progenitor cells. (**d**) Anti-CLL1 CAR-T cytotoxicity on blasts from four different AML patients at an indicated E:T ratio. Data represent the mean values of triplicates +/− SD. (**e**) Anti-tumor activity of anti-CLL1 CAR-T in an HL60 xenograft model. Graph shows quantified tumor burden over time by bioluminescent imaging plotted as individual mice. IgG4SVLVH construct or a control CAR-T were transduced into T-cells of two different donors (CAR expression on day of injection >75–80%) and assayed under the same protocol (*n* = 3 for each donor). Mice injected with PBS or control CAR-T were sacrificed after three weeks due to disease progression.

To explore the activity of anti-CLL1 CAR-T on non-cancerous cells, we evaluated their cytotoxicity and hematopoiesis on healthy CD34+ cells isolated from cord-blood and their cytotoxicity on circulating neutrophils isolated from peripheral blood from healthy donors. We confirmed that CLL1 was detectable at low levels only in the CD34+CD38+ progenitor subset, as previously described [10] (Figure 2a and Figure S2). After 4 hours of incubation with anti-CLL1 CAR-T (E:T = 10:1) we observed no toxicity on CD34+CD38+ cells (Figure 2b), suggesting a minimum threshold on CLL1 expression for CAR-T-cell-mediated killing. We observed a not significant decrease on the granulocyte-macrophage progenitor colonies (CFU-GM) compared to the un-transduced control, while no differences were seen on the other progenitor cell colonies (burst-forming units-erythroid (BFU-E), colony-forming units-granulocyte, erythroid, macrophage, megakaryocyte (CFU-GEMM)) (Figure 2c). For comparison, we generated CD123 CAR-T [22]. This CAR induced robust toxicity on both CD34+CD38− and CD34+CD38+ cells and significantly impaired HSC colony formation, as previously reported [22] (Figure 2b,c). To extend our studies on the side effects associated to target CLL1, we tested the activity of anti-CLL1CART on neutrophils, as granulocytes and monocytes express CLL1 [10]. Accordingly, anti-CLL1 CAR-T induced cytolysis in neutrophils, although it was significantly lower than that induced in HL60 tumor cells (15–39% and >50%, respectively), corresponding with a lower level of CLL1 expression by neutrophils [10] (Figure S3a,b). Neutropenia is a common side effect of anti-cancer therapies and is clinically manageable by prophylaxis with granulocyte colony-stimulating factor and antibiotics [23]. Activated T-cells promote the survival of neutrophils mediated by cytokine secretion [24], and previous studies indicated that neutrophils can induce both pro- and anti-inflammatory effects [25,26]. To rule out the possibility that granulocytes may affect CAR-T activity in this assay, we analyzed the cytotoxicity against HL60 in the presence of neutrophils in a co-culture assay. Cytotoxicity was consistent to our previous findings (Figure S3b). These results suggest that anti-CLL1 CAR-T may preferentially target tumor cells in vivo with limited collateral damage to healthy CD34+ cells or CLL1+ granulocytes.

We next tested the activity of anti-CLL1 CAR-T against primary, patient-derived AML blasts. All samples expressed CLL1 in accordance with previous reports [10] (Figure S4). Interestingly, the stable expression of CLL1 on chemotherapy-resistant leukemic stem cells and blasts during the various stages of the disease suggests that a broad number of patients will benefit from CLL1-targeted CAR-T cell therapy [14]. In this regard, healthy donor-derived T-cells transduced with anti-CLL1 CAR-T effectively killed primary AML blasts with comparable levels of cytotoxicity to those previously observed for HL60 under similar conditions (40–60% and 50% respectively, E:T = 1:1) (Figure 2d). To evaluate the anti-tumor activity of anti-CLL1 CAR-T in vivo, we carried out an aggressive disseminated xenograft model using luciferized-HL60 cells [27], in which mice without treatment die within three weeks of tumor cell inoculation. Two independent donor peripheral blood mononuclear cells (PBMCs) were transduced with anti-CLL1 CART or control CAR-T, and 10 million T-cells (75–80% CAR+) were injected on day 6 after tumor inoculation. Anti-CLL1 CAR-T completely eradicated tumors by day 90 (Figure 2e and Figure S5a). Anti-CLL1 CAR-T demonstrated long persistence in vivo by efficiently controlling disease relapses for 80 days after initial tumor regression (Figure 2e and Figure S5a), a characteristic needed for cancer regression in the clinic [28]. CAR-T-cells were detected in blood but no evident expansion was observed (Figure S5d). Anti-CLL1CART produced pro-inflammatory cytokines 24 h after injection (Figure S5b). We monitored body weights and clinical signs for all models with no signs of toxicity associated to CAR-T-cell treatment (Figure S5c). In this regard, the introduction of suicide genes or antibody-mediated depletion targets into the transduced CAR construct has proven effective in depleting CAR-T-cells after efficient tumor eradication and would represent a safety mechanism in a future clinical setting [11,29].

Together, our results support CLL1 as a highly promising AML target for CAR-T-cell therapy, while potentially reducing the risk of life-threatening side effects, and remark the importance of the hinge selection on CAR design by pointing the shortest construct as the best configuration for our anti-CLL1-CAR, conferring superior activity in vitro and excellent anti-tumor efficacy in vivo.

## 3. Materials and Methods

### 3.1. Cells and Human Derived Samples

MOLM13, MOLM14, and HL60 (ATCC) were cultured as per ATCC recommendations. HL60 cells were luciferized by lentiviral transduction (Firefly Luciferase (FLuc)-IRES-Puro Lentivirus, BioSettia, San Diego, CA, USA). Deidentified primary human AML specimens were obtained from non-treated patients at Scripps Green Hospital La Jolla, after written consent for a protocol approved by the Scripps Institutional Review Board. Blasts were isolated by Ficoll-Paque gradient and cryopreserved in 90% heat inactivated (HI) human serum 10% DMSO. Thawed blasts were cultured overnight in StemSpan FSM (StemCell Technologies, Vancouver, BC, Canada) supplemented with IL-3, GM-CSF, and SCF (Peprotech, Rocky Hill, NJ, USA). Blasts were identified by gating on CD45/SSC (87–95% blasts). Human T-cells were obtained by Ficoll-Paque purification of peripheral blood mononuclear cells from healthy donor whole blood from The Scripps Research Institute’s Normal Blood Donor Service, under The Scripps Research Institute’s Institutional Review Board approval. CAR-T-cell production (lentiviral generation, T-cell transduction, and CAR-T-cell expansion) was carried out as previously described [17]. CD123 CAR-T construct was previously described [22].

### 3.2. In Vitro Assays

Cytotoxicity was measured by flow cytometry. Cells were stained with CellVue Claret (Sigma-Aldrich, St. Louis, MO, USA) or PKH67 (Sigma Aldrich, St. Louis, MO, USA) following the manufacturer’s protocol. CAR-T-cells were deprived of IL-2 for 16 h prior to being incubated for 20 h with target cells, at indicated E:T ratios, in 5% FBS RPMI media (Thermo Fisher Sci., Waltham, MA, USA). Cells were then stained with 7-AAD (BD, Franklin Lakes, NJ, USA) prior to acquisition on LSR-Fortessa X-20 (BD, Franklin Lakes, NJ, USA). Data was analyzed using FlowJo software (TreeStar, Ashland, OR, USA) whereby dead target cells were gated as either APC+ or FITC+ and 7-AAD+. The percentage of cytotoxicity was calculated as ((% dead-target cells)/total % target-cells) × 100. In vitro cytokines were quantified using the Human Th1/Th2 Cytokine Bead Array kit II (BD). CFU assays: HSCs were isolated from human cord blood (San Diego Blood Bank, after obtaining informed consent) using EasySep Human CD34 Positive Selection Kit (StemCell Technologies, Vancouver, BC, Canada). After 4 h of incubation with CAR-T-cells, the mixture was seeded on semi-solid media (StemCell MethoCult SF, StemCell Technologies, Vancouver, BC, Canada) and colonies were scored following the manufacturer’s instructions. Antibodies used: anti-CLL1PE and hFc-block were purchased from BD; anti-CLL1FITC, anti-CD38PE, and anti-CD34APC were purchased from Biolegend (Biologend, San Diego, CA, USA).

### 3.3. Xenograft Model

Female (7–8 weeks old) NOD.Cg-Prkdcscid Il2rgtm1Wjl/SzJ mice (NSG) (Jackson Laboratories, Bar Harbor, ME, USA) were inoculated with 5 × 10^5^ HL60luc IV (Day 0). On day 6, 10 × 10^6^ anti-CLL1 CAR-T-cells, or a control CAR-T targeting a non-related antigen (75–80% CAR+), or PBS were delivered intravenously. Tumor burden was measured by luciferase signal and monitored bi-weekly. Mice were euthanized upon losing more than 15% of body weight or hind limb paralysis. For CAR-T-cell detection, blood was collected at each indicated time point, 50 µL samples were stained for CD3 (anti-CD3BUV395, BD, Franklin Lakes, NJ, USA) and anti-CAR-T (anti-mIgGAPC, Jackson Immuno Research, Baltimore Pike, West Grove, PA, USA), and then they were analyzed by flow. Protocols were approved by the Institutional Animal Care and Use Committee at Calibr.

## Supplementary Materials

Supplementary materials can be found at http://www.mdpi.com/1422-0067/18/11/2259/s1.
