# Supplementary material for: Development of A Chimeric Antigen Receptor Targeting C-Type Lectin-Like Molecule-1 for Human Acute Myeloid Leukemia
Source: Int J Mol Sci. 2017 Oct 27;18(11):2259. doi: 10.3390/ijms18112259 (PMC5713229; doi:10.3390/ijms18112259)

Supplemental Figure 1. Sorting of CAR-T cells results in comparable CAR expression for all constructs. Seven days after transduction CAR-T cells were magnetically sorted with Donkey anti-mIgG-biotin (Jackson Immuno Research) and anti-biotin magnetic beads (Miltenyi Biotec) following manufacturer's protocol and were grown on AIMV media supplemented with 5% human serum and 100IU IL-2. CAR expression was quantified by flow 24 hours after sorting staining with Goat anti-mIgG-APC (Jackson Immuno Research).

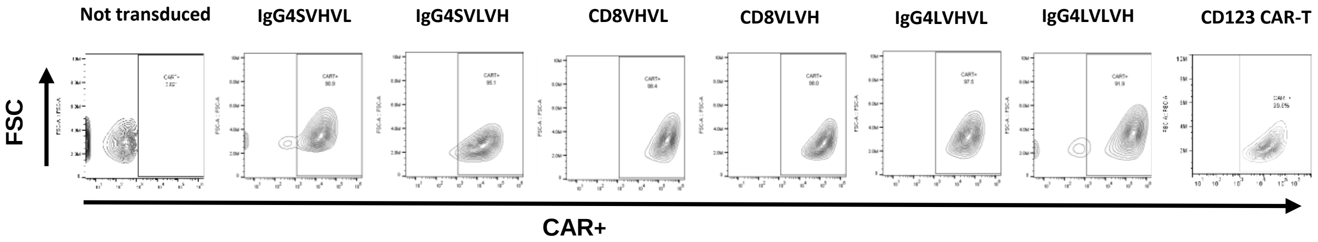

Supplemental Figure 2. Hematopoietic Stem Cell characterization. (a) Gating strategy. **(b)** CLL1 phenotypic plots for each cell subset on FL1-A (CLL1). **(c)** CLL1 expression comparison between HSC populations. Graph shows histogram for each population. Unstained cells and HL60 are included as reference.

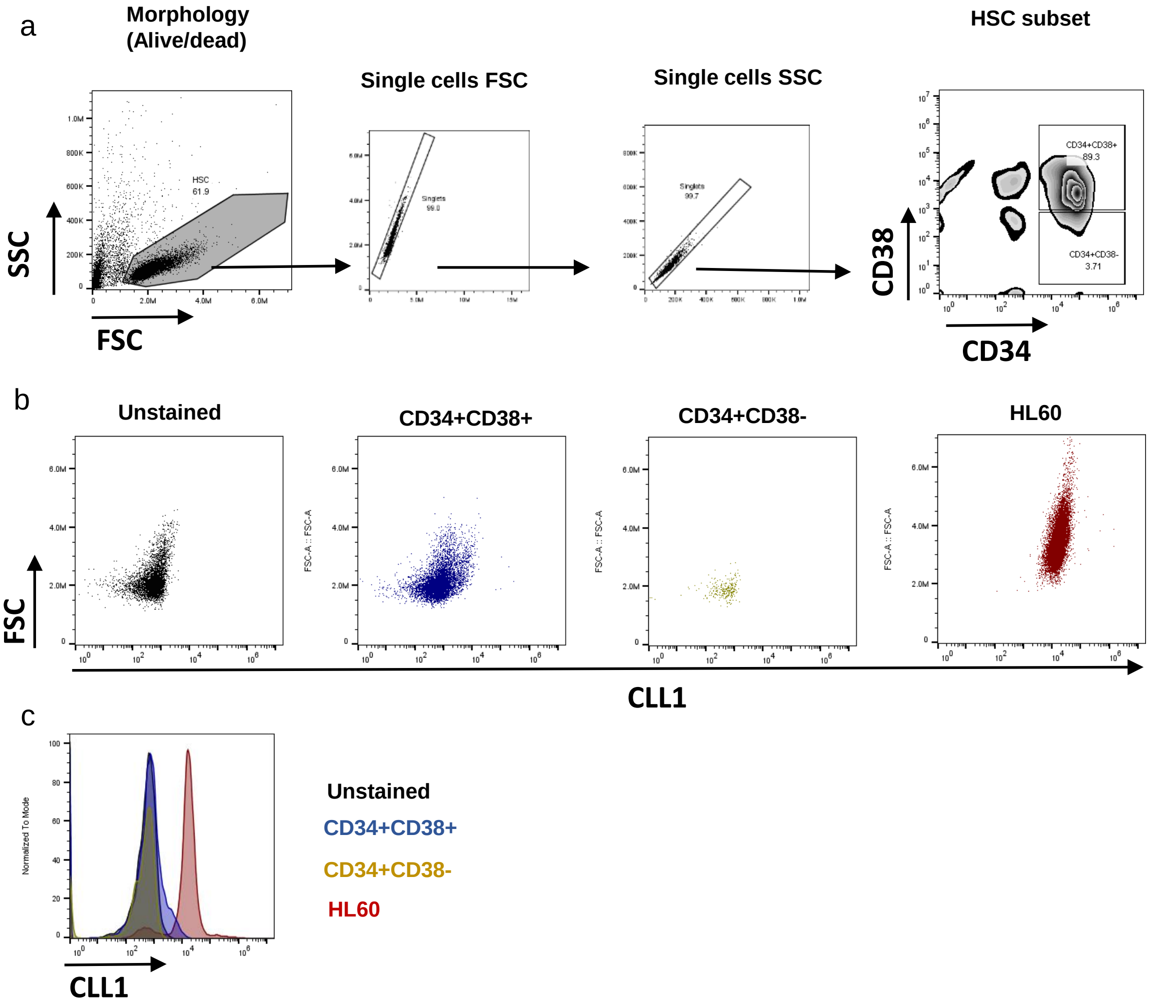

Supplemental Figure 3. Anti-CLL1 CAR-T (IgG4SVLVH) induces cytotoxicity on healthy-donor granulocytes *in vitro*. (a) Healthy donor isolated neutrophils express CLL1. Neutrophils were isolated from healthy donor fresh blood using human MACSxpress Neutrophil Isolation Kit (Miltenyi Biotec) and stained with anti-CLL1-PE (50C1 , BD) and hFc-block (BD). HL60 cells were co-analyzed for comparison. Data shown is representative of 3 different donors. (b) In vitro on-target off-tumor activity. Left columns: anti-CLL1 CAR-T cytotoxicity on neutrophils or HL60 cell line (E:T 5:1) (Data shown is representative of 3 different donors; Neutrophil % cytotoxicity ranged from 15-39%; control CAR-T was used as unspecific killing control showing <2% cytotoxicity, data not shown). Right columns: neutrophils and HL60 cells were co-cultured with anti-CLL1 CAR-T and incubated as per cytotoxicity assays (10:1 E:T) to evaluate the impact of neutrophils on anti-CLL1 CAR-T activity.

a

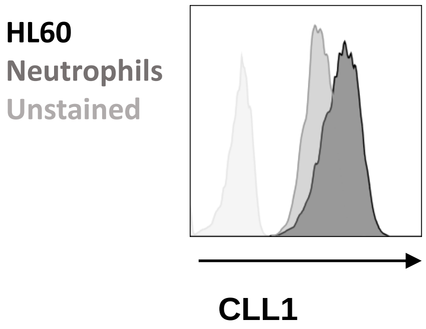

b

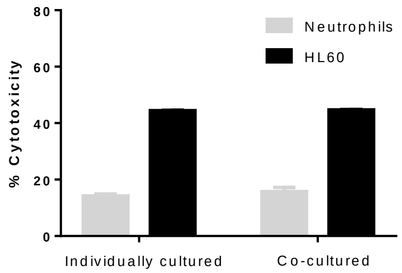

Supplemental Figure 4. Patient derived AML blasts express CLL1. Patient derived AML blasts (dark grey) CLL1 expression. Thawed blasts were stained (anti-CD45PE (Biolegend), anti-CLL1 FITC (Biolegend), hFc-block, (BD)) and analyzed by flow. CD45-SSC gate was used to identify blasts (87-95% blasts) and CLL1-FITC expression compared to unstained controls (grey) for each patient sample.

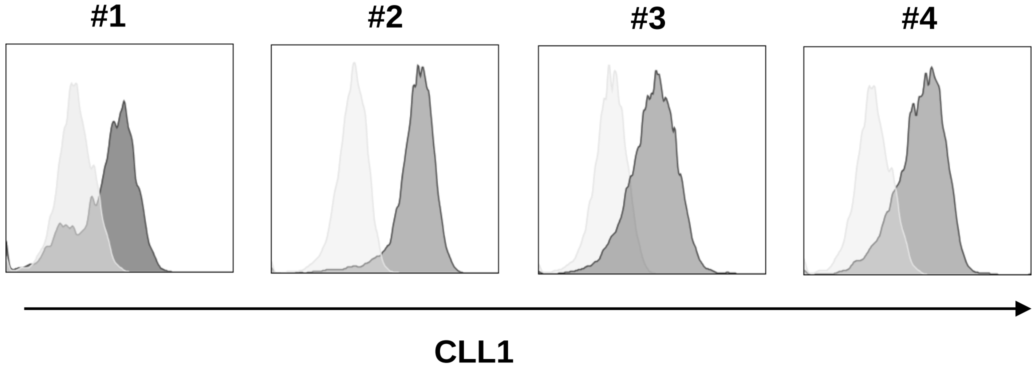

Supplemental Figure 5: (a) Representative BLI images from in vivo efficacy experiments. Female NSG mice were inoculated intravenously with 5 × 10^5 HL60luc and 6 days later mouse (n=3) with same tumor burden were assigned to each group. Tumor burden was measured bi-weekly by IVIS using (IVIS Lumina XR and XenoLight D-Luciferin, PerkinElmer) and was quantified as radiance in the region of interest, which was the area of one mouse. On day 6, 10 × 10^6 anti-CLL1 CAR-T (IgG4SVLVH) (75-80% CAR+), or control CAR-T or PBS were injected intravenously. Mice were euthanized upon losing more that 15% of body weight or hind limb paralysis. (b) Cytokine quantification in serum after 24h of CAR-T administration using V-PLEX Proinflammatory Panel 1 Human Kit (Meso Scale Diagnostics, Rockville, MD, United States). (c) Mouse weights monitored over all in vivo model. Mean +/-SD is shown for each group. (d) CART quantification in blood. Each time point represents the mean +/-SD for each group of CD3/CART+ cells. Blood from not injected mice was used as control after day 12. Protocols were approved by the Institutional Animal Care and Use Committee at California Institute for Biomedical Research.

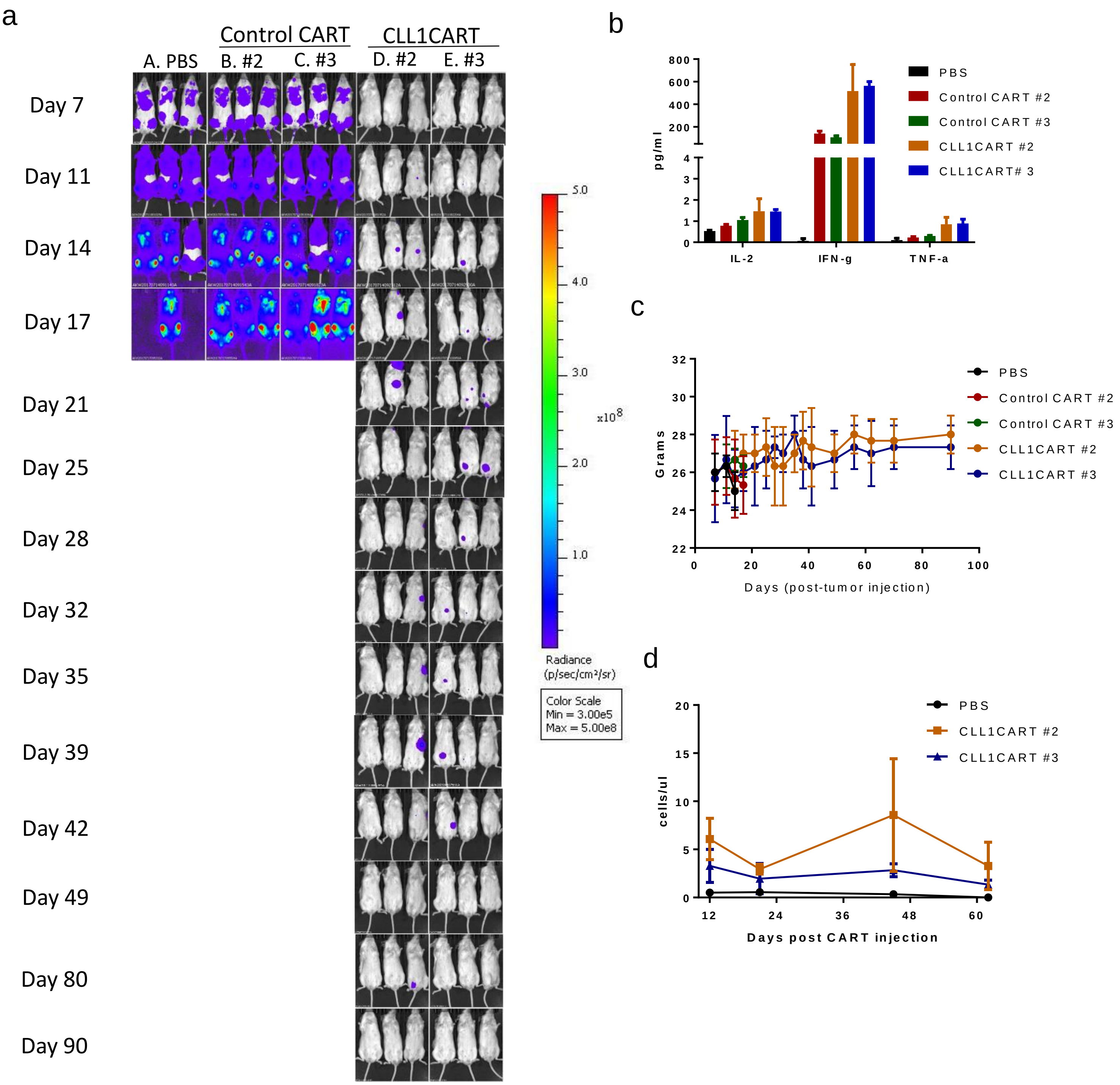

Supplement: Supplementary file 1 [file ijms-18-02259-s001.pdf]
